# Prerequisites and challenges of the role played by death Doula for supporting patients with cancer in end-of-life (EOL) stages: Qualitative study

**DOI:** 10.1371/journal.pone.0343920

**Published:** 2026-06-02

**Authors:** Imane Bagheri, Atena Dadgari, Naiire Salmani

**Affiliations:** 1 Research Center for Nursing and Midwifery Care, Comprehensive Research Institute for Maternal and Child Health, Shahid Sadoughi University of Medical Sciences, Yazd, Iran; 2 Meybod School of Medical Sciences, Shahid Sadoughi University of Medical Sciences, Yazd, Iran; University of Technology Sydney, AUSTRALIA

## Abstract

End-of-life (EOL) doulas support dying people and their families. They are a response to the expectations of healthcare professionals (including palliative care providers) in end-of-life situations.The present study aimed to explore the perspective of palliative care team members about the prerequisites and challenges of the role played by death doulas to support patients with cancer in EOL stages. This qualitative study was conducted in 2023–2024 through in-depth semi-structured interviews with 14 healthcare staff. Participants were selected using purposive sampling considering maximum diversity and the study’s inclusion criteria. Conventional content analysis using Lundman and Graneheim’s approach were applied for data analysis. Data analysis revealed two main themes: the prerequisite for establishing the doula role (categories:drivers for shaping the doula role, recruitment and training of doulas, infrastructural preparation) and challenges ahead (categories:patient/family unawareness and lack of formal recognition). Based on the findings, policymakers and planners can develop operational programs to establish the role of death doulas and, by taking anticipated potential challenges into account, formulate preventive strategies. Furthermore, in light of the identified educational gaps, comprehensive educational content can be designed to adequately prepare death doulas for their roles.

## Introduction

Cancer is a life-threatening disease with a significant global burden. It is the second leading cause of death worldwide [1]. The International Agency for Research on Cancer (IARC) estimated nearly 20 million new cancer cases and 9.7 million cancer deaths in 2022. Demographic-based projections suggest new cancer cases could reach 35 million by 2050 [2]. The high cancer death rate emphasizes the need for EOL care. It also underlines addressing death as a critical issue in cancer care [3].

This is while tday death has not escaped the reach of medicalization. and advances in medical technology,death is increasingly seen as a medical failure to be avoided at all costs. This perspective has led to a growing reliance on life-sustaining treatments and interventions that prolong life,sometimes at the expense of quality [4] and medicalized death often leaves gaps in emotional and spiritual care. Factors like caregiver burnout, lack of training, high workloads, and organizational limits hinder holistic care [3,5]. In other words, current healthcare systems fail to meet the needs of dying patients and their families [6].

In such situations, care needs are often met by family, friends, and social networks [3,5]. Families bear physical, emotional, and financial burdens [7]. They often struggle alone to manage their patients with inconsistent services with limited support from healthcare providers [8]. Moreover, geographic separation and smaller family sizes can leave patients unsupported in their final days [9–11]. This has led families to rely on informal caregivers, like EOL doulas, to fill care gaps [12].

The role of “death doulas” first emerged in Australia [13] and has since expanded to the UK, Canada, and the US [14]. The emergence of this role indicates a potentially significant new response to changing norms, demands, and concerns related to EOL and dignified EOL care [15]. Death doulas have arisen not only as a response to the overwhelming demands placed on families and caregivers but also as a response to the expectations placed on healthcare professionals (including palliative care providers) in EOL situations [16].

EOL doulas serve as companions, advocates, and non-medical guides, supporting individuals at the end of life and their families [17]. They provide a wide range of services, including assistance with advance care planning, ensuring comfort, facilitating religious rituals, physical care, emotional support, and compassion [12,18,19], and addressing the emotional, spiritual, and practical aspects of death [12].

To clearly understand the roles, strengthen collaboration, resolve inconsistencies, and ensure the effective integration of the death doula role into end-of-life care, thereby improving support for patients and families, it is important’to identify the more pressing role implementation needs [19]. On the other hand, the diversity of roles among death doulas and the presence of conflicting and varied perspectives among different professionals regarding the nature and scope of these roles pose a challenge [20].

Moreover, ambiguity in roles [21,22], how to access support and educational programs (21,23), and the absence of a regulatory body governing the conduct of doulasare among the additional challenges highlighted for the engagement of death doulas [23]. Focusing on the novelty of this concept and the lack of precise clarification of roles [24], the associated challenges and required infrastructures [25], qualitative studies grounded in social contexts can identify the challenges and necessary infrastructures for effective performance in this area and contribute to the implementation of best practices for delivering more effective end-of-life care and services [26].

Given the high prevalence of cancer in Iran, palliative care services have been formally implemented for over a decade. Palliative care is now firmly established as an integral component of the national healthcare system, operating under the supervision of the Ministry of Health. This context necessitates the establishment of the end of life doula role, a role whose utility is increasingly being recognized. This need mirrors the recent emergence and acceptance of birth doulas within the iranian healthcare system, particularly in private hospitals, where they provide crucial physical, psycho-emotional, and affective support to expectant mothers during labor and the postpartum period [27].

However, the integration of end of life doulas has not yet materialized formally, unlike their counterparts in childbirth. This stagnation may be attributed to the prevailing socio-cultural and religious milieu of Iranian society, where most patients at the end of life receive care predominantly from family members at home. Consequently, family caregivers shoulder a significant physical, psychological, and emotional burden. In contrast, international evidence suggests that End-of-Life Doulas can play a meaningful supportive role in end-of-life care by addressing the unmet psycho-social, emotional, and practical needs of both patients and their families.

Accordingly, the introduction of such a role presents a novel opportunity to support family caregivers of cancer patients and enhance the quality of life for both patients and caregivers nearing the end of life in Iran. From a health policy and system planning perspective, developing roles analogous to a bereavement companion a term perhaps more sensitive in the Iranian context requires rigorous, evidence based investigation. Identifying the necessary prerequisites for introducing this role and forecasting potential challenges are crucial steps in designing practical, culturally congruent, and sustainable implementation strategies. Therefore, this qualitative study was undertaken to identify and explicate the infrastructural requirements and associated challenges concerning the development of an end of life companion role within the Iranian end-of-life care setting.

## Methods

### Design

This study used a qualitative design conducted with in-depth individual interviews. This study is reported according to Consolidated Criteria for Reporting Qualitative Studies (COREQ) and Standards for Reporting Qualitative Research [28,29].

### Setting

Members of the care team were recruited from palliative care units located in Isfahan, Tehran, Kerman, Mashhad, Qom, and Kashan Provinces.

### Inclusion criteria for all participants

The inclusion criteria were the willingness to participate in the study, a minimum of two years of experience in palliative care, the ability to communicate verbally, and no confirmed psychological disorders under treatment so a total of 14 care team members with experience in providing care for patients with cancer in palliative care units located in Isfahan, Tehran, Kerman, Mashhad, Qom, and Kashan Provinces (selected for their established cancer palliative care centers) attended the interviews. Care team members were invited to attend the interviews, either online or face to face, via email or text message by a member of the research team. All eligible persons invited agreed to participate in the study.

### Exclusion criteria for all paticipants

Paticipants who were unwilling or unable to continue participating in the study for any reason.

### Relationship with participants

No prior relationship was established between the researchers and participants. Participants knew where the researchers worked and the purpose of the research. The three researchers had an interest in the research. NS and IB had previously worked in palliative care research.

### Data collection methods

Data were collected using in-depth semi structured interviews by two researchers (NS and IB).The majority of interviews (n = 10) were completed by the lead researcher (NS) with IB completing a small proportion (n = 4) whilst the lead researcher was on “business trip. NS was experienced in conducting semi-structured interviews and has certificate in fellowship in palliative care also IB had experience in conducting qualitative studies and her research focus was in the field of palliative care.

This study did not provide a predefined interview guide. The two main questions posed were:What prerequisites may be relevant for the role of death doulas?, What challenges might arise in the implementation of death doula roles?

During the interviews, exploratory questions were also asked to elicit more information and to clarify any ambiguities in participants’ statements. At the end of the interviews, participants were invited to add further comments if they wished, and their participation in the interview session was acknowledged. Participants were also informed that if new questions emerged after transcription of the interview content, they would be invited to participate in additional interviews. In this research we tried to address communication impasses in interviews with listen actively, maintain a respectful and calm demeanor, use supportive techniques like rephrasing questions, establish strong rapport by creating a comfortable environment and adjust our interviewing style to the participant’s needs.

### Data analysis

The interview texts were analyzed using qualitative content analysis as described by Graneheim and Lundman [30]. Three researchers with diverse backgrounds in data analysis participated: NS, a professor and PhD supervisor who holds a fellowship in palliative care and is a seasoned qualitative researcher with numerous publications in reputable journals; IB, a PhD supervisor and researcher in palliative care and qualitative studies; and AD, a researcher in palliative care and qualitative studies who is a doctoral student. The principal investigator (NS) prepared the interview texts after each interview. All researchers read the interview texts repeatedly to become familiar with the overall content. Two members of the research team, NS and IB, identified meaningful units, summarized them, and assigned codes to them. The third researcher (AD) read and analyzed the interview texts independently. NS and IB continuously compared codes and semantic units to identify similarities and differences, and they re-examined the raw data when ever necessary. The codes were then organized into subcategories and categories. The entire analysis process was conducted through reflection and discussion until consensus was reached between the two researchers. In case of disagreements, consultation with the third researcher (AD) occurred. The final categories were discussed among the research team. Coding and data analysis were performed using MAXQDA-2020. The results were emailed to some participants and asked to indicate their agreement with the categories and subcategories: “I fully agree with these finding.”

### Transparency and trustworthiness of findings

To enhance data credibility, after conducting the interviews and completing the initial coding, the study findings were reviewed and revised by some participants based on their comments. To ensure the reliability of the findings, in addition to the researchers, several experts in qualitative research (peer review) were invited to examine the findings and assess the rigor of the data analysis process. To further ensure the trustworthiness of the findings, an external auditor reviewed the codes and extracted categories and compared them against a sample of interview excerpts, making revisions based on the feedback provided by the auditor. To improve transferability of the findings, the results were shared with several experts who did not participate in the study, who compared and aligned the findings with their own experiences. Finally, to examine confirmability, the themes and categories extracted by some external raters who did not participate in the study were reviewed and confirmed.

### Researchers’ positionality

We actively practiced reflexivity. Reflexivity included consciously examining our own beliefs, assumptions, and methods, and how these unconsciously influence the study design and the data analysis process [31]. To further support active reflexivity, we employed strategies such as awareness of our own interests and values, and our over-arching assumptions as researchers—through immersion, use of prompts, and regular reflection and review of these elements.

### Ethical approval

The protocol used in this study was approved by the Ethics Committee of Shahid Sadoughi University of Medical Sciences, Yazd with the code of ethics IR.SSU.REC.1402.004. After receiving verbal consent and completing the written consent form participants, they were involved with study and to maintain anonymity, the interviews were conducted only in the presence of the participants and the researcher(s), and the confidentiality of the information and audio files was maintained. The participants were also reminded that they would be free to withdraw from the study at any time. At the end of the study, the researcher’s phone number was provided to the participants so they could be informed of the study’s results if desired.This research were conducted from 25/4/2023–29/7 2024. In this study, participation was completely voluntary and participants were not paid as a reward for their participation, and they were assured that the research findings would be made available to them for study and use if they so desired.

## Results

The participants in this study were 14 care team members with experience in providing care for patients with cancer in palliative care units One psychologist, one psychiatrist, one psychiatric nurse, one social worker, three faculty members with fellowship or experience in palliative care, one spiritual counselor, two oncologists, and four oncology nurses. In one case, a follow-up interview was conducted to clarify and expand on certain responses, but no additional follow-ups were deemed necessary for other participants during the analysis.Each interview typically lasted 45–60 minutes.

The mean age of participants was 45 years and 57.14% of them were female. Most of the participants (42.9%) had 15–20 years of professional experience. Half of the participants held doctoral degrees, while the other half held bachelor’s or master’s degrees. Participants shared their perceptions regarding establishing the role of death doulas in EOL cancer care.

**Fig 1:**
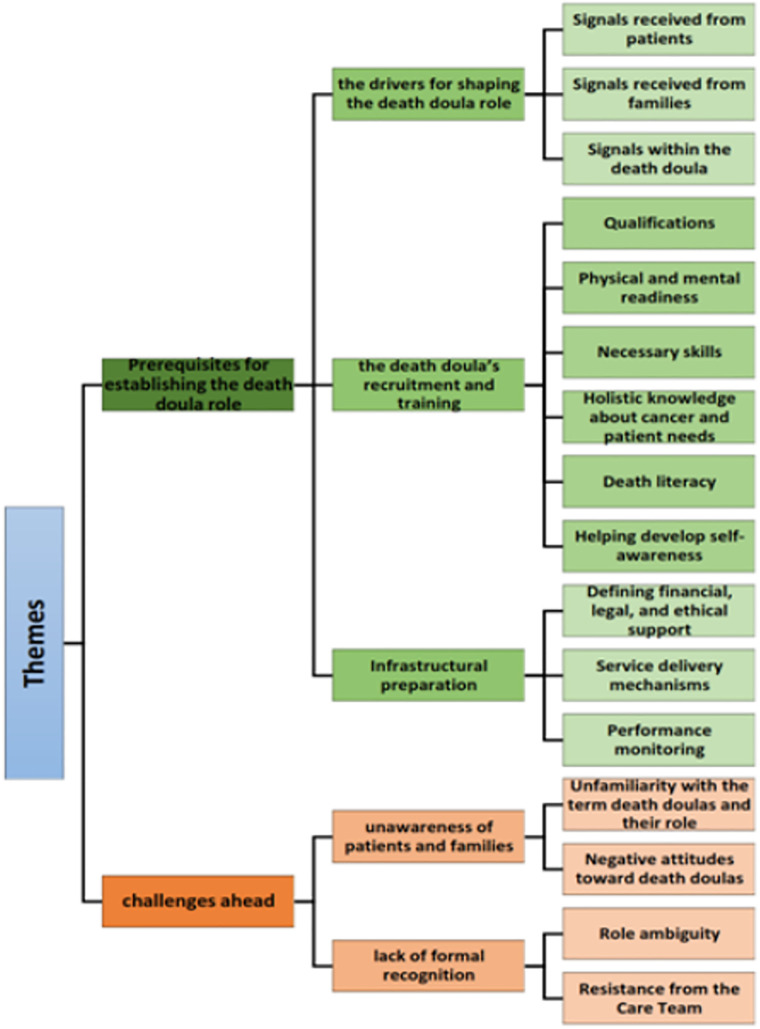
Themes, Category, Subcategory.

The data analysis yielded 118 codes, 16 subcategories, five categories and two main themes. These themes—prerequisites for establishing the death doula role and challenges ahead—are presented in Fig 1 and Theme, Category, Subcategory, Code and Quotations are presented in Table 1.

**Table 1:** Theme, Category, Subcategory, Code and Quotations.

Theme	Category	Subcategory	Code	Quotations
Challenges ahead	1. Patient/family unawareness	1. Unfamiliar terms and role	1. The absence of the concept of the word “doula” 1. Unawareness of the expected duties of a doula 3. Ambiguity in understanding the term “doula” 4. Equating a doula with other palliative care providers	“The reality is that neither patients nor even the healthcare staff are aware of this role. This is my first time hearing the term, even though informally. We perform many of the duties of a doula for our patients and their families” (Nurse 2)
		2. Negative attitudes toward death doulas	5. The negative connotation of the term “death doula” 6. Exploitation of people’s death by a doula 7. A doula’s interference in the work of doctors 8. Becoming a doula for financial gain	“I don’t have a very positive view of death doulas. The association of the word doula with death carries a lot of negative connotations, and it would be better to find a better name, like companion or supporter” (Psychiatrist) “You’ve seen how negative people’s views are about those who work in morgues as if making money from the process of death is somehow distasteful. I think the same view might exist towards death doulas” (Psychiatric Nurse)
	No formal recognition	3. Resistance from the healthcare team	9. Continuing ineffective treatments 10. Refusal to accept doulas as colleagues in the team 11. Preventing referral of patients to doulas 12. Hostility toward doulas 13. Viewing doulas as competitors	“In my opinion, as long as oncologists do not stop ineffective treatments such as chemotherapy, radiation therapy, and surgeries until the final days, death doulas won’t be able to practice End-of-life (EOL)care” (Nurse 3) “I think death doulas won’t be able to function as they should due to the prevailing atmosphere in hospitals and the treatment-focused mindset of hospitals and doctors” (Nurse 2)
		4. Role ambiguity	14. Unclear location of doula activities 15. Ambiguity in the work process 16. Uncertainty in how the role integrates into the system 17. Uncertainty about the doula’s role in communications	“The problem is that we’ve only set up one oncology department in the hospitals, but we don’t know many things” (Nurse 3) “The work process, job description, and the place of service for death doulas need to be clearly defined; otherwise, we know that these ambiguities can be a major obstacle to implementing the doula role” (Faculty Member 1)
Prerequisites for establishing the doula role	1. Doula recruitment and training	1. Qualifications	18. Minimum academic qualifications related to medical sciences 19. Member of the healthcare team 20. Nurse Assistant 21. Nurses 22. Psychologist aware of spiritual issues 23. Experience working with cancer patients 24. Non-professional individual with at least essential competencies 25. Having lived experience 26. Family member	“Maybe a death doula is someone who has experience in caring for cancer patients and can understand what the patient and their family need to support them. There have been times when we had a patient in the ward whose caregiver wasn’t feeling well, so we’d call one of the mothers of our previous patients, who had passed away, and ask, ‘Would you come by? We have someone here we can’t communicate with, and the person won’t accept their patient, even the psychologist can’t convince them.’ The person would come, talk with them, and share their experiences” (Nurse 2) “My experience over all these years shows that if a doula is part of the healthcare team, they can be much more helpful” (Faculty member 2) “A death doula can be a member of the patient’s family, their close friends, or someone who has specialized training, knows how to speak to them, and knows how to provide care” (Oncologist) “If we give the knowledge of death doulas to a caregiver, I might say they’re the best person to play this role” (Faculty Member 3)
		2. Mental and physical preparedness	1. No serious disability or impairment 2. Physical ability to perform basic care tasks such as changing positions 3. No negative view of death 4. Calmness 5. No psychological disorders 6. Problem-solving ability 7. High adaptability 8. Ability to cope with burnout 9. Hopeful	“A doula must have peace within themselves. Someone who is stressed cannot bring calm to their audience” (Psychiatric Nurse 1) “I think a death doula requires a strong personality, as not just anyone can play this role” (Nurse 4) “In my opinion, a doula should have experience working with cancer patients. Another important issue is that they should undergo tests and be psychologically certified. They should be mentally healthy, without personal mental health issues, and not be on medication” “These individuals should have at least basic psychological training and possess a high ability to adapt” (Psychiatrist 1)
		3. Required skills	36. Responding to patient and family questions 37. Holistic assessment of the patient and family 38. Gaining trust 39. Establishing communication 40. Spiritual care 41. Psychosocial care 42. Assisting in basic nursing procedures 43. Providing counseling 44. Physical care	“A doula must know basic care tasks, such as when to change a catheter, how to empty it, and how to perform a gavage” (Nurse 4) “A doula must recognize the symptoms that may arise for the patient and know how to manage them. For example, what to do if the patient has a fever, or if they experience shortness of breath, what actions to take” (Oncologist 1) “In my opinion, the first important point is that a doula must have the skill to gain the trust of the patient and their family, know how to communicate with them, and then address their physical, emotional, and psychological problems” (Psychiatrist 2)
		4. Holistic knowledge about cancer and patients’ needs	45. Possible causes of patient discomfort 46. Somatic symptoms of anxiety 47. Common physical problems 48. Hidden pain symptoms 49. Factors influencing pain expression (cultural background, emotional state, cognitive function, etc.) 50. Patient’s concerns 51. Social 52. Cultural 53. Emotional-psychological 54. Recognizing spiritual needs 55. Awareness of the difference between religion and spirituality	“To provide effective care, a doula must have strong knowledge about spiritual, psychological, and physiological aspects of death” (Psychiatric Nurse) “One issue is that many of our patients don’t report their pain. I don’t know, maybe in their culture, expressing pain isn’t acceptable. The doula must understand this issue and we should teach them what symptoms could indicate pain. Pain is very important. Cancer patients experience a lot of pain in their final days, and we need to know how to alleviate it” (Nurse) “A doula must have psychological and mental health knowledge. For example, they should know how to manage patients when they’re feeling hopeless, depressed, or anxious, and how to prepare them for death using various techniques” (Psychologist) “In my opinion, spiritual care in the final days is extremely important. Patients become more focused on their spiritual needs as they approach death. The doula should understand that spiritual beliefs vary among individuals, and their needs differ accordingly. Understanding this issue can help them provide better care” (Spiritual Counselor)
		5. Death literacy	56. Understanding the stages of confronting the reality of death by Kubler-Ross 57. Understanding the coping mechanisms with death by Kubler-Ross 58. Familiarity with the rites and rituals of the dying person based on religion 59. Familiarity with mourning ceremonies based on religion 60. Familiarity with the process of writing a will following religious guidelines 61. Understanding and recognizing the anxiety and fear of death in the patient 62. Understanding the process of death	“A patient goes through a multi-step process before accepting death, which in psychology we call the Kubler-Ross’ five stages. To put it simply, when patients realize their death is near, they first deny it, then become angry, then bargain with God to live longer, then become depressed, and eventually accept death. The doula must understand this process and know how to behave with the patient at each stage” (Psychiatrist 1) “Patients have customs and rituals before and after death depending on their culture and religion. The doula must know these traditions and accompany them through these rituals” (Social Worker)
		6. Developing one’s worldview	63. A positive outlook on life 64. A positive outlook on the afterlife 65. Understanding one’s values regarding life and death 66. Understanding one’s philosophy of life 67. Analyzing one’s attitude towards death and dying 68. Awareness of the afterlife 69. Being aware of one’s own religious beliefs and convictions	“An important point is that the doula must first reflect on themselves, considering what values are important to them and what are not and what are their religious beliefs about death and life. Once they recognize these issues, they’ll be clear about their purpose and what their mission is for this role” (Psychologist).
	2. Infrastructural preparation	1. Determining legal, financial, and ethical support mechanisms	70. Determining legal aspects 71. Determining rights and benefits 72. Determining ethical aspects 73. Support from doctors and officials 74. Support from non-governmental organizations (NGOs) 75. Support from insurance	“Working with patients in the final stages may expose the doula to ethical challenges. As a nurse, I sometimes have doubts about what is truly right. Should we perform CPR on the patients or not? If we don’t do it, will we face legal issues? If we do CPR and the patient recovers, what’s the point if they end up passing away again tomorrow?” (Nurse 3) “This role can only be successful if insurance organizations support it. Otherwise, if patients are expected to have a death doula at home and pay for it themselves, the cost will undoubtedly be high, and they may decide to forgo it” (Social Worker)
		2. Determining the service delivery mode	76. Providing service as an extra staff during shifts 77. As an alternative 78. Voluntarily 79. Starting from the hospital and following up at home 80. With prior consent and coordination with the patient and family 81. Visitation sessions, not continuous presence 82. In-person sessions 83. Short sessions 84. Providing service in any location (home, hospice, hospital)	“The death doula’s service to the patient and family should start from the hospital and continue at home after discharge. This is, in my opinion, the best scenario” (Nurse 2) “The family must give formal permission to start the doula’s work. A doula cannot just go to the patient’s home and perform the role without their family’s permission” (Psychiatric Nurse) “In my opinion, there is no need for the doula to be always present at the patient’s bedside. The presence should be regular, but as a visit rather than full-time” (Oncologist 1)
		3. Specifying doula performance monitoring methods	85. Supervising the doula’s performance 86. Evaluating the outcomes of doula performance 87. Post-training evaluation to assess qualifications 88. Issuing accredited certificates 89. Conducting required training sessions	“The knowledge of the death doulas should be updated. What I mean is, we shouldn’t train them and then leave them; instead, we should periodically organize update courses for them” (Faculty member 3) “Death doulas, after entering the field, should be evaluated for the reliability and consequences of their work” (Faculty Member 1) “Death doulas must acquire the necessary qualifications, pass exams, be evaluated, and be prepared before starting their work. These should be outlined in the training courses” (Faculty member 3)
	3. Drivers for shaping the doula role	1. Signals received from the patient	90. The importance of caring for the dying person, not the corpse 91. The importance of a humanistic approach to End-of-life (EOL)care 92. The inhumanity of dying alone 93. No one should die alone 94. Psychological harm to the patient 95. Denial of death 96. Death in solitude without support 97. Spending the hard days in the final stages of life 98. Passive euthanasia 99. Suitable for solitary patients 100. The needs of end-stage patients 101. The patient’s approach to the final stages of life 102. The high cost of ineffective treatments 103. Death is inevitable 104. The ineffectiveness of treatment 105. Frequent hospital visits by the patient 106. Performing futile treatments in the hospital	“In the past, when a patient was dying, they were placed in a room alone, away from others, to pass away... but now things are different. End-of-life (EOL)care for patients has become very important. I read somewhere that no human being should die alone” (Nurse 1) “A lot of patients have children who have gone abroad, and when they become ill, there’s no one to care for them. They experience difficult days and moments, and no one helps them” (Nurse 2) “Patients and families mostly prefer that the patient stays in the hospital to receive care there. So, the patient is admitted to the oncology ward, and some procedures are carried out for them, but passive euthanasia may occur when the nurses don’t pay attention to the medication treatments and instructions and don’t carry them out” (Faculty Member 1)
		2. Signals received from the family	107. Family needs 108. Family’s feeling of loneliness at home 109. The destabilization of the family’s situation 110. The vicious cycle of family stress on the patient 111. Psychological harm to the family 112. Family fatigue 113. Family disintegration	“Sometimes, the patient’s family gets exhausted and stops cooperating. At first, everyone visits the patient and surrounds them, but after a while, no one comes anymore, and only one person stays, carrying the entire burden of care and this causes them to run out of energy” (Nurse 2) “Sometimes, family members get into conflicts with each other in these situations because they are under intense stress, and the slightest trigger makes them lash out at each other” (Faculty member 1) “There are times when the doctor tells the family that nothing more can be done for the patient, and they should take the patient home to spend their final days there. When the patient is taken home, the family feels more alone because there’s no one to carry out the care, and they don’t know how to do it themselves..” (Faculty member 2)
		3. Internal signals from the doula	114. Manifestation of doula’s beliefs in behavior and actions 115. Spirituality 116. Belief in the positive impact of the role on personal life 117. Interest 118. Seeing the essence and philosophy of one’s life in this role	“As an oncology nurse, sometimes I ask myself, ‘You’re the person who is here to play this role. Why did I choose to become a nurse, come to a foreign city, and study while having a small child?’ It’s one of the purposes of my life” (Nurse 3) “Our creation is not aimless. Perhaps I came here to play this role at a specific moment in time and place” (Nurse 3) “The beliefs of a doula should be reflected in their actions, not just in appearance. They must be visible in their behavior because families are smart and will quickly notice it. Otherwise, the family will not trust them” (Spiritual Counselor) “Oncology nurses say the work we do here has a positive impact on our lives. For example, we had a nurse who couldn’t have children. After a few years, she got pregnant. She says, ‘I worked here, and the prayers of the families were with me, and that’s why I got pregnant.’ Our problems could have almost broken us, but they haven’t, and it’s because of this belief we hold” (Nurse 1)

### Theme 1: Prerequisites for establishing the death doula role

The prerequisites for establishing the death doula role theme include three categories. Categories include the drivers for shaping the death doula role,the death doula’s recruitment and training and the death doula’s recruitment and training.

**1.1 The drivers for shaping the death doula role**

The drivers shaping the death doula role category include 3 subcategorries: the signals received from patients, signals received from families, and signals within the death doula:

**1.1.1 Signals received from patients:**

The majority of participants highlighted the pressing realities such as the high cost of ineffective treatments, the inevitability of death, the importance of caring for a dying person rather than just the body, and the humanistic approach to EOL care underscore the need for the death doula role, particularly for terminally ill patients without caregivers or those facing loneliness. Observations such as nearing the end of life, denial of death, emotional and psychological distress, and enduring hardships during the final days amplify the importance of this role.

**1.1.2 Signals received from families:** Several participants emphasized that families also require support during EOL care. Without such support, families may experience exhaustion, loneliness, and psychological trauma, leading to familial instability and potential disintegration. This family stress can cyclically affect the patient, worsening their physical and mental state. Therefore, training death doulas to support families during these critical times is crucial.

**1.1.3 Signals within the death doula:** number of participants stated that a spiritually inclined doula, motivated to assist patients, who finds meaning and purpose in this role, would be more effective. Believing that the role positively impacts their personal life and integrating their beliefs into their actions could facilitate the doula’s active participation.

**1.2 Death doula’s recruitment and training**

Death doula’s recruitment and training category included 6 subcategories: qualifications, physical and mental readiness, necessary skills, holistic knowledge about cancer and patient needs, death literacy, and self-awareness:

**1.2.1 Qualifications**

Many participants stated that individuals selected for this role should have at least an academic background related to medical sciences and be a member of the healthcare team, such as nurses, nurse assistants, or clinical psychologists with experience in working with cancer patients. Some participants suggested that death doulas could also be non-professionals with the necessary capabilities, such as family members or individuals with lived experiences in this area.

**1.2.2 Physical and mental readiness**

The selected individuals must undergo assessments to ensure their physical and mental readiness. Doulas need sufficient physical strength to provide basic care, such as repositioning patients, and should not have severe disabilities. The participants described ideal doulas as calm, optimistic, problem-solving, and highly adaptable individuals. They should also be free from psychological disorders and possess a positive perspective on death to support patients and families effectively.

**1.2.3 Necessary skills:** Training is essential for individuals assuming the death doula role. Several participants stressed the importance of communication skills to establish trust with patients and families. Doulas should assess patients’ and families’ needs and provide comprehensive care across physical, psychological, social, and spiritual dimensions. They must address patients’ and families’ questions and concerns, offering appropriate counseling. In addition, having nursing skills, could help avoid improper hospitalizations.

**1.2.4 Holistic knowledge about cancer and patient needs:** The majority of participants emphasized that training should encompass holistic knowledge about cancer and its impact on patients. Doulas need to understand common physical issues, such as underlying causes of symptoms, hidden pain indicators, and factors influencing pain expression (e.g., cultural background, emotional state, and cognitive function). They should recognize signs of anxiety and somatization and provide effective emotional care. Furthermore, doulas must understand patients’ spiritual needs and the distinction between religion and spirituality in patients to offer better spiritual care. Considering the humanistic approach adopted by death doulas, cultural and social factors of patients and their families should also be taken into account.

**1.2.5 Death literacy:** An essential component of training death doulas is enhancing their death literacy, which included understanding the process of dying, patient anxiety and fear of death, the stages of coping with death as per Kübler-Ross’s framework, and effective coping mechanisms. Doulas should also be familiar with the rituals and customs of dying individuals, mourning ceremonies, and the preparation of wills following religious guidelines.

**1.2.6 Helping develop self-awareness:** Death doula training should include opportunities to help doulas develop self-awareness regarding their worldview. The participants emphasized that doulas need a positive outlook on life and the afterlife, clarity about their values, beliefs, and religious perspectives on life and death, and an understanding of their philosophy of life. They should also analyze and reflect on their attitudes toward death and dying.

**1.3 Infrastructural preparation**

Infrastructural preparation category included 3 subcategories: defining financial, legal, and ethical support, determining service delivery mechanisms and monitoring doula performance.

**1.3.1 Defining financial, legal, and ethical support:** Many participants emphasized the importance of establishing ethical, legal, and compensation frameworks before introducing death doulas into patient care. Sustaining this role requires the support of physicians, administrators, insurance providers, and NGOs.

**1.3.2 Service delivery mechanisms:** After training, plans should be developed for how death doulas will provide services. The participants suggested these services could be offered voluntarily, on a short-term or sessional basis, rather than as continuous involvement. Services could be provided in various settings (home, hospice, or hospital). In hospitals, doulas could begin as auxiliary staff during shifts and later, with prior coordination with the patient and family, continue care at home after patient discharge.

**1.3.3 Performance monitoring**

Before commencing work, death doulas should be assessed for competency, certified, and subsequently supervised. Regular evaluations should be conducted to identify areas for further training and improvement. Moreover, the outcomes of their services should be systematically monitored.

### Theme 2: Challenges ahead

The challenges for implementing the doula role theme include 2 categories: unawareness of patients and families and lack of formal recognition.

**2.1. Unawareness of patients and families**

Unawareness of patients and families category include 2 subcategories: unfamiliar with the term death doulas and their role and negative perceptions of death doulas.

**2.1.1. Unfamiliarity with the term death doulas and their role:**

The majority of participants highlighted that the term “doula” is unfamiliar to the Iranian public, as it lacks cultural and linguistic roots in the country. unfamiliarity with the concept of doulas represents a key gap in comprehending their purpose. The participants also pointed out that The juxtaposition of “death” and “doula” may evoke confusion, as the word “death” carries heavy connotations that might not align with the supportive nature of the role. the presence of “death” in the term often leads to a cascade of questions: “Isn’t death the end of life? Who is a death doula? What does a death doula do?” and Ultimately, the unfamiliarity with the term “doula”, limited understanding of the role and responsibilities of death doulas, the absence of this concept in the Iranian cultural context, and insufficient awareness of the importance of such a role may all contribute to public misconceptions.

**2.1.2 Negative attitudes toward death doulas**

Several participants acknowledged that the focus on death doulas intervening only during the final stages of life and engaging with patients and families before and after death could create the impression that doulas exploit others’ grief to secure a livelihood. Essentially, the death of an individual might be seen as an opportunity for income generation or for death doulas to showcase their role and attract clients. The participants also pointed out that part of the negative perception surrounding death doulas comes from the healthcare team, especially physicians, who view doulas as intruders in the treatment process. They perceive the presence of doulas in the final stages of life as unwelcome, believing that doulas hinder doctors from continuing their treatment efforts. Another issue highlighted by the participants was the belief that the emergence of the doula role is merely a way to make money. It was suggested that the role is designed to capitalize on the final moments of life and serves as a minimal-effort job for those lacking any other form of employment or business, allowing them to generate income relatively easily.

**2.2 Lack of formal recognition**

Lack of formal recognition category include 2 subcategories: Resistance from the Care Team and Role ambiguity

**2.2.1 Resistance from the Care Team:**

The acceptance of doulas as a member of the treatment team was another issue highlighted by participants. They suggested that the treatment team typically consists of individuals with diverse specializations who collectively form a unified circle aimed at advancing the treatment process and caring for the patient. For death doulas to play a role within this framework, the circle must expand to include a new member—a death doula—who can begin their role in the EOL stages. However, this integration cannot occur until the treatment team becomes open to the presence of doulas.

A lack of acceptance would mean no opportunity to define or fulfill the doula’s role. Another problem was the reluctance of medical staff to refer the patient to death doulas even at the final stages of life or when treatments are deemed ineffective. In such situations, medical staff are often reluctant to allow doulas to provide support to dying patients and their families. This lack of acknowledgment in the care environment hinders doulas from performing their role effectively, as it disrupts their interaction with patients and families.

The presence of doulas could challenge the continuation of futile treatments and emphasize care that ensures peace and comfort in a patient’s final days. This shift may position doulas as individuals who redirect patients and families away from traditional treatment approaches. The philosophy and type of services offered by doulas differ from conventional medical practices, and if these services gain acceptance, it might foster a sense of rivalry among other care team members.

**2.2.2 Role ambiguity:**

The ambiguous position of death doulas within the healthcare system can be considered a fundamental challenge to their ability to perform their role. If the position of doulas and the scope of their work within the healthcare system remain undefined, their role will lack clarity, hindering their integration. Similarly, if the specific settings where doulas are to operate are not established, and, most importantly, if the process of integrating this role into the system is not adequately clarified, death doulas will not be able to function effectively or gain practical applicability.

## Discussion

This study explored the perspective of experts, including care team members and health educators, regarding the establishment of the role of death doulas. An analysis of the participants’ perceptions indicated that the implementation of the death doula role to support EOL patients and their families requires fulfilling certain prerequisites, such as “drivers for shaping the doula role”, “recruitment and training of doulas”, and “infrastructural preparations”. Furthermore, challenges such as “patient/family unawareness” and “lack of formal recognition” were highlighted in this process.

### Prerequisites for establishing the doula role

Prerequisites for establishing the doula role arise from recognizing signals arising from the patient, the family, and the dual-role itself. These three elements are interconnected, like the three sides of a triangle, in which the emergence of one side stimulates the emergence of the others. In line with this finding, Silva and colleagues highlighted indicators such as worsening health conditions, rapid decline in function, frequent and unplanned hospitalizations, reduced responsiveness to treatments, and a higher burden of complex symptoms as signs signaling approaching end of life. They emphasized that early prediction of caregiving needs leads to better planning and improved end-of-life care [32].

According to participants statements, as patients enter the terminal stages of life, their dependence on family members increases so their family feel a heavy caregiving burden and they need the presence of an individual to support them in the EOL stages. In support of this finding, Rawlings and colleagues state that the presence of ‘dual-role’ can be associated with a reduction in death-related fear and anxiety, empowerment of families, and the provision of psychological, emotional, and spiritual support [16].

The findings also showed that the internal signals of the doula were part of the “drivers for shaping the doula role”. Intrinsic motivators such as a desire to help others, spirituality, interest in EOL care, and a search for the meaning of life drive individuals toward the doula role. Aligning with this finding, Claxton highlighted the “aha moment” experienced by doulas, where becoming a doula is not a linear process but often stems from prior work in palliative care or hospices. During this moment, they realize their purpose—to help others, educate them, and promote the acceptance of death as a natural part of life despite its sadness [33]. The pursuit of emotional and spiritual fulfillment as motivators for becoming a doula is similarly noted in studies by Guirguis (2008) and Claxton (2011) [33,34]. Krawczyk and Rush (2020) also explored doula’s perspectives, finding that some chose the role after caring for a dying individual and recognizing the need for better support and education for other patients and families during this process [15].

Another prerequisite for establishing the doula’s role is recruitment and training. this category require assessing several factors: the applicant’s educational background, whether they are part of a healthcare team, a volunteer interested in the doula role, a family member of the patient, or someone with caregiving experience for dying individuals, as well as their physical and mental readiness for the role.

The literature provides limited information on these criteria. For instance, Lentz’s study suggested that doulas can be experienced palliative care nurses who are active, retired, or semi-retired [35].

Elliott (2011) highlighted that doulas are trained mentors or volunteers who provide non-medical services [36]. Rawlings et al. (2020) stated that doulas with a nursing background can conduct comprehensive assessments and collaborate with care teams to manage the dying individual’s symptoms. However, this dual role of being both a doula and a healthcare team member may create role overlap, leading to confusion. For example, families might expect medical care from doulas with healthcare experience [16].

Another criterion identified in the present study was having experience in caring for a patient during the dying process. Consistent with this finding, Rawlings et al. (2020) noted that some doulas attributed their decision to become death doulas to their prior professional experiences. They had witnessed individuals who did not experience a “good death” and, as a result, were motivated to provide knowledge and support for others going through the dying process [16].

The participants acknowledged that doulas must receive some training on communication skills, basic nursing skills, death literacy, and worldview development. Tzeciak-Kerr’s (2016) study identified the topics covered in an intensive doula training course, including preparing for the doula role, self-care, the dying process, cultural influences, finding meaning, spirituality, religion, and active listening skills [37].

The final “prerequisite for establishing the doula role” was infrastructural preparation. Accordingly, for death doulas to function effectively, foundational elements must be established, such as determining how doulas will be supported, how services will be provided, and how their performance will be. Krawczyk et al (2020), in line with this finding, stated that regarding licensure, standardization, and professionalization of the dual-role across various domains, there are diverse perspectives, and inconsistency within legal frameworks sometimes leads to inadequate training programs. Therefore, quality control and oversight of performance were emphasized as essential to ensure the competence of the dual-roles and the issuance of appropriate licenses [20].

Another issue that must be clarified in any setting where the role of a doula is being implemented is compensation methods. During an informal survey at a 2022 symposium, doulas attended from seven countries, predominantly from Australia, Canada, the United States and the United Kingdom, various perspectives on this topic were discussed, including the use of sliding scale criteria, payment through donations or contributions, payment via private health insurance systems, payment through subsidies defined in the care system, and finally, the possibility of fulfilling the doula role voluntarily and without financial compensation [20]. Rawlings et al. (2023) surveyed 12 doula training organizations and reported that six organizations opposed the idea of doulas working for free. They argued that if doulas work on a volunteer basis, it could devalue the role of doulas as trained professionals. They emphasized that compensation should be established to value the services provided by doulas. Four organizations stated that whether or not to charge for services should depend on an agreement between the doula and the client (the patient and their family) [38].

Another aspect that needs to be clarified within the framework of service provision is the method of service delivery by death doulas. In a review of the literature, the only study addressing this issue was conducted by Lentz (2014), which suggested that meetings between death doulas, patients, and families could be scheduled regularly based on changing circumstances, establishing a consistent pattern of interaction (daily, weekly, monthly, or via phone calls) [35].

### Challenges ahead

The data in the present study showed that the challenges for implementing the doula role are unawareness of patients and families (unawareness of doulas and their roles and negative attitudes toward death doulas) and lack of formal recognition (resistance from the healthcare team and unknown position).

Since the role of death doulas has been established and recognized in European countries but is still emerging in Iran, its acceptance might be influenced by Iranian cultural norms and many other factors. The unfamiliarity of the term “doula” within Iranian culture, combined with the negative connotations associated with the word “death”, can create an adverse impression for listeners. Besides, the ambiguity surrounding this concept hinders its rapid and easy acceptance. Those attempting to introduce this role within the care system may find themselves entangled in a web of unanswered questions from service recipients.

In this context, Krawczyk et al. (2020) have argued that while there is widespread agreement on the importance of naming the role, there is also a wide range of perspectives on standardizing the terminology. Many doulas do not find the term “end-of-life doula” to be ideal. This is not solely due to cultural issues but also because of the general public’s limited understanding of the word “doula” and the power dynamics that standardizing names and practices entails. Among these terms, “end-of-life doula” and “death doula” are becoming the most common. They broadly encompass a specific range of supportive, emotional, and spiritual services provided before and after death, which can vary significantly across regions and countries. In a study by Rawlings, doulas mentioned that they act as additional support for dying patients and emphasized the need for public awareness of their role. They stressed that as a unified community, they must educate the public to enable informed decision-making [15].

The second challenge identified in this study was the lack of formal recognition of the doula role caused by “resistance from the care team” and “role ambiguity”. The unclear status of doulas in society can pose challenges to their role. Determining whether doulas are part of the public healthcare system or operate within private hospitals, palliative care centers, or hospices is crucial, as the designated status affects how services are delivered and how doula practices integrate into the country’s care system. The ambiguity regarding the status of doulas also impedes effective collaboration between care teams and doulas. For instance, physicians might be reluctant to refer patients to doulas, resulting in the lack of acceptance of doulas in healthcare teams and the continuation of futile treatments.

Consistent with these findings, a study conducted by Torki Harchegani et al. (2024), which aimed to identify the challenges associated with employing birth doulas in Iran, revealed significant psychological barriers. Midwives reported feeling that Birth Doulas were overstepping their boundaries and attempting to usurp the responsibilities of the midwifery staff. Specifically, delivery room midwives often perceived Birth Doulas as inefficient individuals incapable of assisting the treatment team [39]. Furthermore, the research by Azimi Lolaty and Rezaie Abhari documented role ambiguity concerning the duties of Birth Doulas as a major impediment cited by stakeholders. This lack of attention to their defined roles, coupled with their unspecified legal status, acted as a significant barrier to the effective performance of the birth doula role [40].

In line with this finding, Yoong et al. (2022) stated that the role of doulas remains unclear to both the general public and professionals, which may stem from the variations in doula roles across countries and settings. The failure to understand the role of death doulas can hinder their integration into palliative and EOL care services. Confusion about their role makes collaboration between clients and care team members with doulas challenging. Some doulas might even encounter hostility from medical teams during their service, due to concerns over overlapping roles, perceived threats, or the absence of official regulations governing doula practices. In some cases, doulas might face disregard [19]. In Rawlings’ study, many doulas expressed their desire to ensure that medical professionals did not perceive them as competitors or threats [16].

Krawczyk et al. also identified the integration of death doula’s activities within palliative care or hospice centers as a concern. They raised the challenge of aligning the services provided by doulas with those of these centers [38]. In a similar vein, Rawlings et al. noted that many movements related to EOL doulas today are focused on achieving public recognition and integration into healthcare [25].

### Strengths and limitations of the study

The present study provided valuable new knowledge about healthcare team members’ perceptions of death doulas in Iran. As the first study of its kind, it explored the prerequisites and challenges associated with the role played by death doulas from the perspective of healthcare professionals. The findings from this study can contribute to the development of new attitudes and improvements in care practices within this field. However, the findings should be considered within the context of the existing limitations. While the data collected are significant, their generalizability is not assured. This limitation stems from our limited access to palliative care centers nationwide. Given that these centers have only been established for about a decade, the number of active professionals and staff remains insufficient. These limitations could lead to selection bias in participant recruitment for the study. Additionally, due to similarities in attitudes and policies related to palliative care, the diversity of viewpoints gathered may not be sufficiently broad. This issue may affect the interpretation and analysis of results and indicates the need for more extensive research in the future. Future studies should employ diverse approaches and a broader geographical range to gather data to achieve a more comprehensive and accurate understanding of the issue and contribute to the improvement of care policies and practices. Future research should extend this study to include additional participants, such as patients, family members of patients, and oncologists, in order to capture diverse perspectives across different contexts. This broader understanding can inform the development of practical efforts to support the implementation of death doula’s roles. Summarized based on the opinion of the second referee.

### Implications for practice/research/education

**Practice**: Findings from this study could be used to initiate the role of death doulas as guides. Policymakers and planners, relying on identified needs and established infrastructures, can develop operational programs to implement this role. Taking into account the potential challenges identified in this study, they can formulate preventive strategies through collaborative deliberation among end-of-life care specialists.

**Research:** Based on the results of this study, subsequent complementary research can be designed qualitatively. By conducting interviews with death doulas, the perspectives of these practitioners regarding their most pressing needs for fulfilling their roles can be understood. Through a comparative study between the views of experts and those of death doulas, differences and similarities can be identified, and the findings from these studies can be applied in practice to improve the quality of role performance.

**Education:** Another application of the current study’s results lies in education. Educational planners can use the identified educational gaps from this study to develop the content of a death doula’s curriculum, thereby designing a comprehensive and practical program that supports death doulas in performing their roles effectively.

## Conclusions

The analysis of attitudes among health care team members working in palliative care showed that the successful implementation of the death doula role to support cancer patients in the final days of life requires identifying the factors shaping the doula role, preparing the necessary infrastructure, and recruiting and training doulas. efforts suggests that directed toward establishing the required infrastructure, including: determining legal, financial, and ethical support mechanisms; determining the service delivery mode; and specifying doula performance monitoring methods. These foundations are essential for shaping and establishing an appropriate environment for the deployment of death doulas, thereby paving the way for the education of death doulas.

During the development of this role, its training, and the deployment of death doulas within end-of-life care, two key challenges may arise: patient/family unawareness and lack of formal recognition. This lack of awareness and recognition of the existence, purpose, and functioning of death doulas can hinder the fulfillment of the doula’s role.
